# Intraoperative Anterior Segment Optical Coherence Tomography in the Management of Cataract Surgery: State of the Art

**DOI:** 10.3390/jcm11133867

**Published:** 2022-07-04

**Authors:** Mario Damiano Toro, Serena Milan, Daniele Tognetto, Robert Rejdak, Ciro Costagliola, Sandrine Anne Zweifel, Chiara Posarelli, Michele Figus, Magdalena Rejdak, Teresio Avitabile, Adriano Carnevali, Rosa Giglio

**Affiliations:** 1 Eye Clinic, Department of Public Health, University of Naples Federico II, 80131 Naples, Italy; toro.mario@email.it; 2 Chair and Department of General and Pediatric Ophthalmology, Medical University of Lublin, 20079 Lublin, Poland; robert.rejdak@yahoo.com; 3 Eye Clinic, Department of Medicine, Surgery and Health Sciences, University of Trieste, 34134 Trieste, Italy; tognetto@units.it (D.T.); giglio.rosam@gmail.com (R.G.); 4 Eye Clinic, Department of Neuroscience and Reproductive and Odontostomatological Sciences, University of Naples Federico II, 80131 Naples, Italy; ciro.costagliola1957@gmail.com; 5 Department of Ophthalmology, University of Zurich, 8091 Zurich, Switzerland; sandrine.zweifel@usz.ch; 6 Department of Surgical, Medical and Molecular Pathology and of Critical Care Medicine, University of Pisa, 56126 Pisa, Italy; chiara.posarelli@med.unipi.it (C.P.); michele.figus@unipi.it (M.F.); 7 Faculty of Medicine, Medical University of Warsaw, 02091 Warsaw, Poland; rejdakmagdalena@gmail.com; 8 Department of Ophthalmology, University of Catania, 95124 Catania, Italy; t.avitabile@unict.it; 9 Department of Ophthalmology, University Magna Graecia of Catanzaro, 88100 Catanzaro, Italy; adrianocarnevali@unicz.it

**Keywords:** anterior segment OCT, intraoperative OCT, cataract surgery, surgical technique

## Abstract

Background: The introduction of non-invasive diagnostic tools in ophthalmology has significantly reshaped current clinical practice in different settings. Recently, different anterior segment (AS) intraoperative optical coherence tomography (i-OCT) systems have been employed for different interventional procedures including cataract surgery. Materials and Methods: A review on the use of AS i-OCT in the management of cataract surgery, following the Preferred Reporting Items for Systematic Reviews and Meta-Analyses guidelines (PRISMA). The level of evidence according to the Oxford Centre for Evidence-Based Medicine (OCEM) 2011 guidelines, and the quality of evidence according to the Grading of Recommendations Assessment, Development and Evaluation (GRADE) system were assessed for all included articles. Results: Out of 6302 articles initially extracted, 6302 abstracts were identified for screening and 32 of these met the inclusion/exclusion criteria for full-text review; 19 articles were excluded. Conclusions: The use of AS i-OCT in cataract surgery, even if only a few studies have a high level or grade of evidence, may represent a useful tool for novel surgeons approaching phacoemulsification but also for expert ones for teaching purposes and to plan and manage complicated cases.

## 1. Introduction

Cataract surgery is one of the most cost-effective healthcare interventions. It affects both physical and psychological health [1,2] and it has undergone a significant modernization in the past fifty years [3]. Indeed, this procedure has been made effective and safe thanks to the introduction of minimally invasive techniques and the availability of innovative equipment.

Recently, intraoperative optical coherence tomography (i-OCT) systems have been integrated into ocular microscopes, providing useful feedback for the surgeons of both the anterior and posterior segments of the eye [4,5].

I-OCT is a non-invasive, real-time method with high resolution that can image the finest ocular structures even through mediums with significant opacity. However, to date, the extent of the actual benefits of the application of i-OCT into common clinical practice is still debated [6].

This review aims at summarizing the current applications of anterior segment (AS) i-OCT in the management of cataract surgery while assessing the level and quality of the studies included in the review.

## 2. Materials and Methods

This systematic review was conducted and reported by the Preferred Reporting Items for Systematic Reviews and Meta-Analyses (PRISMA) guidelines [7]. The review protocol was not recorded in the study design, and no registration number is available for consultation. The methodology used for this comprehensive review consisted of a systematic search of all available articles exploring the use of AS i-OCT in patients undergoing cataract surgery. A literature search of all original articles published up to November 2021 was performed in parallel by two authors (MDT and MW) using the PubMed database.

The following terms were employed for “Cataract Extraction” (Mesh) OR “Refractive Errors” (Mesh) OR “Cataract” (Mesh) OR “Lens Implantation, Intraocular” (Mesh) OR “Anterior Eye Segment” (Mesh) AND “Tomography, Optical Coherence” (Mesh).

Furthermore, the reference lists of all identified articles were examined manually to identify any potential study not captured by the electronic searches. After the preparation of the list of all electronic data captured, two reviewers (MDT and MW) examined the titles and abstracts independently and identified relevant articles. Exclusion criteria were review studies, pilot studies, case series, case reports, photo essays, and studies written in languages other than English. Moreover, studies performed on animal eyes, cadaveric eyes, and pediatric patients were excluded as well.

The same reviewers registered and selected the captured studies according to the inclusion and exclusion criteria by examining the full text of the articles. Any disagreement was assessed by consensus, and a third reviewer (MB) was consulted when necessary. No effort was made to contact the corresponding authors for further unpublished data. All selected articles were analyzed to assess the level of evidence according to the Oxford Centre for Evidence-Based Medicine (OCEM) 2011 guidelines [8], and the quality of evidence according to the Grading of Recommendations Assessment, Development and Evaluation (GRADE) system [9].

## 3. Results

**Figure 1:**
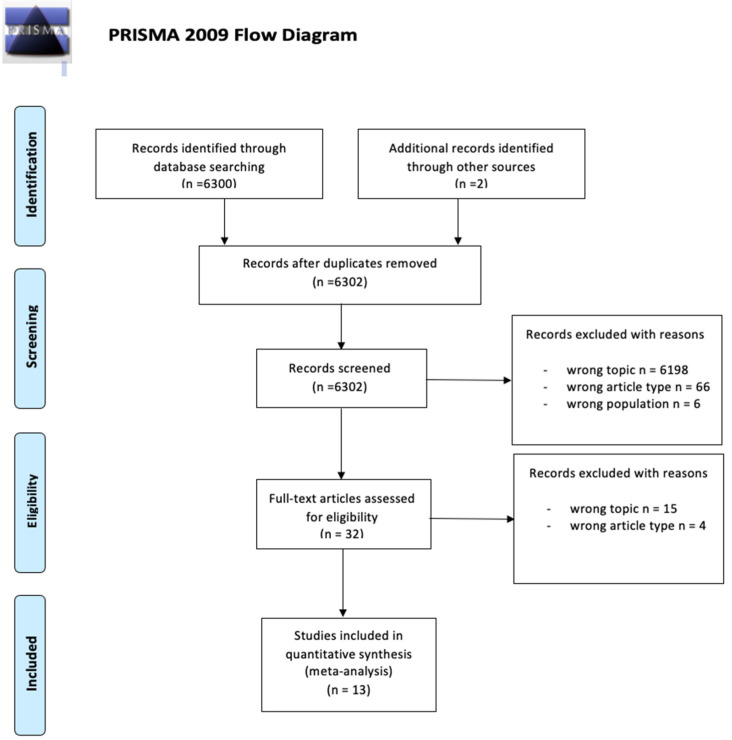
Flow diagram of the study according to Preferred Reporting Items for Systematic Reviews and Meta-Analyses (PRISMA) guidelines [7].

The results of the search strategy are summarized in Figure 1. From 6302 articles extracted from the initial research, 6302 abstracts were identified for screening and 32 of these met the inclusion/exclusion criteria for full-text review. Nineteen articles were excluded (Figure 1).

Studies’ characteristics, main results, level, and grade of the available evidence about the role of AS i-OCT in cataract surgery management are summarized in Table 1.

No data synthesis was possible for the heterogeneity of available data and the design of the available studies. Thus, the current review reports a qualitative analysis, detailed issue-by-issue below narratively.

The i-OCT visualization of ocular changes occurring during cataract surgery (both standard and femtosecond-laser-assisted using LenSx Laser System -Alcon Laboratories) was described by Das et al. [10]. The i-OCT employed was the RESCAN^TM^ 700 (Carl Zeiss Meditec), which is a 3 dimensional (3D) spectral-domain OCT (SD-OCT) characterized by a wavelength of 840 nm, an axial resolution of 5.5 μm, and an A-scan depth of 2000 μm. Continuous video monitoring permitted assessment of wound morphology (length, breadth, number of planes, epithelium disruption, amount of wound gape, endothelial alignment, Descemet membrane detachment-DMD) and wound closure adequacy (corneal stroma whitening and thickening induced by hydration of the side port and the main incision was qualitatively estimated), to visualize capsulorhexis, to clearly monitor hydrodissection and hydrodelineation procedures. Interestingly in two out of three posterior polar cataracts (PCCs), the opacity could be clearly distinguished from the posterior capsule and a safe hydrodissection was performed. Moreover, they applied i-OCT to image the distention of the capsular bag during intumescent cataract surgery, to decide the exact depth of trenching, and to assess the amount of wound distortion during intraocular lens (IOL) implantation, to check the final IOL position.

Tañá-Sanz et al. studied four AS parameters obtained intraoperatively with the i-OCT integrated into the Catalys (Johnson & Johnson Vision) femtosecond laser platform, which is an AS SD-OCT characterized by a central wavelength of 820–930 nm and an axial resolution of <30 μm [11]. The parameters included in the analysis were anterior chamber depth (ACD), central corneal thickness (CCT), lens thickness (LT), and white-to-white (WTW). They compared these parameters with those acquired preoperatively with two swept-source OCT (SS-OCT) biometers (IOLMaster 700-Carl Zeiss Meditec- and Anterion-Heidelberg Engineering). Statistically significant differences were shown for all parameters, with the Catalys being associated with the greatest values of ACD (mean difference with Anterion: +0.183 ± 0.056 mm; mean difference with IOL Master 700: +0.250 ± 0.054 mm), CCT (mean difference with Anterion: +32.110 ± 9.347 μm; mean difference with IOL Master 700: +24.473 ± 10.897 μm) and LT (mean difference with Anterion: +0.026 ± 0.024 mm; mean difference with IOL Master 700: +0.088 ± 0.029 mm) and the shortest WTW (mean difference with Anterion: −0.236 ± 0.604 mm; mean difference with IOL Master 700: −0.385 ± 0.575 mm).

Waring et al. used Catalys’ i-OCT to study possible correlations among ACD, LT, lens diameter (LD, the distance from the intersections of the anterior to posterior lens surfaces), and lens volume (LV, the volume of the lens calculated from the measured anterior and posterior lenticular surface curvatures that were extended to intersect in the lenticular periphery) [12]. While ACD and LT could be easily detected, LD and LV could be acquired only by i-OCT and were related to lens aging. It was found that LV had a strong positive correlation with both LT and LD; all three lens anatomy parameters demonstrated a positive correlation with age (moderate for LT and LV, weak for LD). ACD showed a moderate inverse correlation with LT, a weak positive correlation with LD, and a weak inverse correlation with LV. Biometric data obtained with IOL Master 500 (Carl Zeiss Meditec) were also included. AL had a weak correlation with LD, a weak inverse correlation with LT, and no correlation with LV. The authors provided regression equations to predict LD and LV from conventionally available parameters (AL, ACD, LT, age, average WTW, and average keratometry).

Hirnschall et al. studied if intraoperative lens capsule position after crystalline lens removal could represent a useful parameter to predict IOL position [13]. A capsular tension ring (CTR) was introduced in all patients to cause a taut and straight planar posterior capsule. They used a prototype of an AS time-domain OCT (Visante-Carl Zeiss Meditec-, characterized by a wavelength of 1310 nm and an axial resolution of 18 um) combined with an operating microscope. During surgery, four screenshots were taken: at the beginning of surgery, after irrigation/aspiration (I/A) of cortical material and Ophthalmic Viscosurgical Device (OVD) removal, after implantation of a CTR, and at the end of surgery. Intraoperative parameters were combined with ACD values acquired preoperatively, 1 hour after surgery, and three months postoperatively with PCI meter (Carl Zeiss Meditec), IOLMaster 500, and ACMaster (Carl Zeiss Meditec). Regarding immediate postoperative ACD, the position of the anterior capsule post-CTR insertion (CTRa) was associated with the highest variable importance projection (VIP), followed by the position of the anterior lens capsule after lens removal without a CTR, whereas the posterior lens capsule was a poor predictor. Regarding 1 hour after surgery ACD, it was found that AL and CTRa were excellent predictors, preoperative ACD was good, while LT was poor. AL, CTRa, and preoperatively measured ACD turned out to be excellent predictors of three-month after surgery ACD, while LT was a poor predictor.

In their study, Kurosawa et al. reported a case of posterior capsule dehiscence induced by misdirected laser irradiation, caused by the detection of a high OCT intensity area in the anterior vitreous (misinterpreted as the posterior capsule) [14]. They consequently proposed a method to avoid this complication, called LT inspection: it stands for comparing pre-operative LT (detected with IOL Master 700 and CASIA2, TOMEY) with intraoperative LT (detected with Catalys’ i-OCT) before laser irradiation and, eventually, to manually correct intraoperative data based on this comparison. A total of 546 patients underwent LT inspection: in one case, an inappropriate posterior capsule line was shown, and LT inspection avoided its break. Additionally, 474 patients were retrospectively analyzed, and it was found that four patients (including the previously mentioned case of posterior capsule dehiscence) had an inappropriate posterior capsule detection. However, the “posterior capsular safety margin” (which is a laser setting) of 500 μm avoided the complication in three out of four patients.

Palanker et al. developed a system combining a frequency-domain OCT (FD-OCT, characterized by an axial resolution of 11 mm) with a femtosecond laser system [15]. They made a comparison of laser capsulotomies and manual capsulorhexis in terms of size (measured along the x and y axes, repeated after rotation by 45°) and shape (the circularity was measured as a ratio of the sample area to the area of a disk with a diameter corresponding to the greatest linear dimension of the sample—this ratio is equal to 1 in an ideal circle). Measurements were obtained during surgery right after the capsular disk removal (with a Seibel Rhexis ruler), on the extracted capsule (after removal, they were put between glass slides, stained with 0.5% trypan blue, and then digital light microscopy was performed), and based on the digital images obtained during slit lamp exam one week and one month after surgery. Deviation from the intended size was −282 ± 305 μm in manual capsulorhexis and 27 ± 25 μm in laser capsulotomy. Concerning circularity, they calculated a 0.77 ± 0.15 ratio for manual capsulorhexis, and a 0.95 ± 0.04 ratio for the laser ones.

Titiyal et al. applied the i-OCT integrated on RESCAN^TM^ 700 to study the correlation between morphological characteristics of clear corneal incisions (CCIs) and the incidence of intraoperative DMD, comparing conventional phacoemulsification to femtosecond laser-assisted cataract surgery (FLACS) [16]. Firstly, CCIs internal slit openings were classified by a single surgeon under the operating microscope, at the beginning of surgery before the occurrence of DMD, as ragged slit (RS, irregular wavy appearance) or smooth slit (SS, SS-like uniform appearance). RS morphology was observed in 31.2% of cases of the conventional surgery group and in 13.5% of cases of the FLACS group. Then, incision sites were assessed after incision creation, after phacoemulsification, after irrigation-aspiration, after IOL insertion, and after stromal hydration: DMD was described when Descemet membrane separation from the underlying stroma was visible on i-OCT or both i-OCT and the operating microscope. Forty-three out of 129 cases experienced localized incision-site DMD; incidence was significantly higher in cases with RS morphology (87.1%) than SS morphology (16.3%). All DMDs detected by i-OCT were also detectable under the operating microscope before stromal hydration: however, only i-OCT could detect an increase in its size or its onset after stromal hydration (which represented the phase in which the higher rate of DMD occurred-83.7% cases). Incision sites were also checked one day and thirty days after surgery using slit-lamp biomicroscopy and AS-OCT (RTVue-100; Optovue): at day 30, incision-site DMD wasn’t detectable in any case.

Song et al. analyzed the location of the pupil center (PC), the limbal center (LC), and the lens center (which can be extrapolated from the location of anterior and posterior lens capsule lines) with Catalys’s i-OCT in patients undergoing FLACS [17]. Angle K (consequently, the location of the visual axis-VA) was acquired preoperatively with OPD scan III (Nidek). Lens center-LC distance was 0.205 ± 0.104 mm, lens center-VA distance was 0.296 ± 0.198 mm, while lens center-PC distance was 0.147 ± 0.103 mm (the smallest one); the LC was located significantly inferiorly and temporally compared to the PC. In regards to distances from the VA, the PC had a distance of 0.283 ± 0.161 mm, the LC of 0.362 ± 0.153 mm, and the lens center of 0.296 ± 0.198 mm.

In their study, Mastropasqua et al. studied capsulorhexis features after FLACS and manual cataract surgery [18]. Enrolled patients were randomly divided into three groups: patients of the first group underwent FLACS with Lensx platform (high-definition OCT visualization system), and for the second group Lenstar (Lenstar) FLACS was applied (which is guided by an integrated 3D confocal structured imaging system), while the standard manual technique was used for the last group. Regarding capsulotomy circularity, in the first seven days after surgery it was statistically significantly better in laser groups, but no statistically significant differences were observed at 30 days and 180 days. At all time points, the manual capsulorhexis area was significantly smaller than the laser one. Laser capsulotomies were also associated with a statistically significantly lower deviation from the intended size. In regards to the distance between the pupil centroid and IOL centroid, it was statistically significantly lower in laser groups than in the manual group; the distance between the pupil centroid and capsulotomy was also statistically significantly lower in laser groups than in the manual group.

Titiyal et al. analyzed morphological features and intraoperative behavior of white cataracts with RESCAN^TM^ 700 i-OCT [19]. Regarding surgical steps, capsulorhexis was done under a cohesive OVD (starting with a 26-gauge bent needle cystotome and ending using a micro forceps or needle cystotome based on the intraoperative characteristics). Bimanual I/A of cortical material was needed if an impending risk of capsulorhexis escape was detected. Difficulties in capsulorhexis were subjectively assessed by the operating surgeon based on the surgeon’s control over the size and circularity of the rhexis while performing the anterior capsular flap tear. After gentle hydrodissection, nuclear emulsification and eventually I/A were performed, ending with IOL implantation. Four kinds of cataracts were described: type I (characterized by regularly organized cortical fibers), type II (with a more convex anterior capsule and multiple intralenticular clefts), type III (in which the convexity of the anterior capsule and the clefts were combined with areas of homogeneous ground glass appearance), and type IV (in which the anterior lens cortex had a homogeneous ground-glass appearance). Type I underwent uncomplicated capsulorhexis; in type II cataracts, i-OCT showed a cortical bulge in the anterior chamber during initial nick creation, standing for raised intralenticular pressure with a high risk of rhexis extension and leading the surgeon to perform a bimanual I/A till its lowering; regarding type III and type IV, the lowering of intralenticular pressure was observed at the beginning of the rhexis, outlining an increased risk of extension.

Titiyal et al. also analyzed morphological features of PPCs during standard phacoemulsification by using RESCAN^TM^ 700 i-OCT [20]. At the beginning of surgery, i-OCT was used to assess the morphology of PPC (observed as a hyperreflective region in the posterior pole area), the relation of the opacity to the posterior capsule (which appears as a continuous hyperechoic concave line limiting the posterior aspect of the nucleus) and integrity and continuity of the posterior capsule. After hydrodelineation, the relation between posterior capsule and epinuclear cushion was valued to notice any posterior capsule-epinucleus fluid interface causing accidental hydrodissection. Three types of PPCs were consequently described: type I was characterized by an intact posterior capsule visualized along the entirety of the posterior polar opacity, with rly d from the capsule; in type II, the dense central region of PPC was apparently adherent to the posterior capsule, which could be detected only in the periphery; in type III the posterior capsule status couldn’t be analyzed at all. The preoperative AS-OCT features correlated with the i-OCT features. Type I underwent gentle hydrodissection in addition to hydrodelineation and intraoperative posterior capsule break never occurred; type II and III underwent just hydrodelineation. Accidental hydrodissection occurred in 1 PPC type II during hydrodelineation; however, the posterior capsule remained intact till the end of surgery. Moreover, the incidence of capsule dehiscence in these i-OCT-guided surgeries (7.5%) was also retrospectively compared to not i-OCT-guided surgeries (11.1%) and statistically significant differences were noted.

Anisimova et al. studied the Berger space during and after 15 FLACS and 13 standard phacoemulsifications [6]. Videos were recorded immediately after IOL implantation (through the application of i-OCT integrated on RESCAN ^TM^) and in the early postoperative period (with the RTVue XR 100, Optovue). Berger space was detected in 75% of cases intraoperatively and in 82% of cases postoperatively; in 32% of cases postoperatively, it was occupied by hyperreflective spots and particles, while i-OCT turned out to be more sensitive (57% of cases).

Juergens et al. described the potential benefits of i-OCT during 29 surgical procedures, among which four were cataract surgeries [21]. They employed EnFocus Ultra-Deep OCT (Leica Microsystems) integrated into the microscope, characterized by a maximum penetration depth of 11 mm, a maximum axial resolution of 9 μm, and a maximum lateral resolution of 15–31 μm. The authors described the case of a patient requiring additional implantation of a two-part, brown iris diaphragm because of post-neuro-borreliosis maximum pupillary rigidity, stating that only i-OCT imaging could assess iris diaphragm position in the capsule sac because of the poor contrast between the anterior lens capsule margin and the brown implant.

## 4. Discussion

The introduction of i-OCT-integrated surgical microscopes might represent a further step toward a safer and more efficient surgery. To date, different i-OCT systems can be integrated into an ocular microscope, providing useful feedback for the surgeons both the anterior and posterior segment surgeons [22,23].

When dealing with AS procedures, this technology provides direct visualization of anatomic structures before, during, and after surgical maneuvers, allows for an analysis of surgical planes, guides surgical steps, and helps to detect intraoperative complications, eventually impacting surgical decision-making [5,24,25].

In their study, Tañá-Sanz et al. demonstrated that some AS parameters (ACD, CCT, LT, and WTW) obtained with the AS-SD-OCT integrated into the Catalys femtosecond laser platform differed from those derived from SS-OCT biometers (IOLMaster 700 and Anterion biometer). According to the authors, these differences could be related to patients’ position and the mydriasis required for the surgery [11].

In addition to these parameters, which can be easily acquired by traditional biometry, a greater understanding of lens anatomy (including the dimensions of the aged crystalline lens and its capsule) could be useful for surgeons and the development of new IOL formulas and technologies. However, most studies have been conducted in research settings, applying customized devices and not commonly available instruments (such as Magnetic Resonance Imaging), consequently, results couldn’t be easily applied to clinical practice. Moreover, the ability of more commonly available biometric data to predict LD and LV is quite limited. The introduction of i-OCT integrated on femtosecond laser platforms has facilitated the study of lens anatomy in larger data sets making new lens parameters, consequently, available [26]. In their work, Waring et al. showed that i-OCT could detect LV, LD, and LT and they provided regression equations to predict LD and LV from conventionally available parameters. The authors stated this additional info could help in effective lens position (ELP) estimate/ion (consequently improving IOL power calculation and enhancing refractive predictability) and, in new IOL technologies development, such as capsule refilling [12].

Indeed, the prediction of the IOL position after surgery still represents one of the main issues when dealing with IOL power calculation [27,28].

Hirnshall et al. analyzed if intraoperative lens measurements, instead of preoperative ACD measurements, could improve ELP evaluation. It was found that the position of the anterior capsule after the insertion of a CTR represented an excellent predictor of ACD before surgery. However, it must be stated that to acquire these values, the use of a CTR was required (which is not an ordinary step in uncomplicated surgery) and that preoperative AL and ACD were also associated with high VIP for the prediction of ACD measured three months after surgery [13].

In cataract surgery, i-OCT might represent a valid device both for standard phacoemulsification procedures and for FLACS. Its main applications include the visualization of corneal incisions and the stromal hydration, the assessment of hydro-dissection, perception of the trenching depth, and identification of lens positioning [10,29]. Thus, the use of i-OCT might allow a safer surgical procedure, decreasing the rate of postoperative wound leak and hypotony and preventing any iatrogenic capsular rupture during hydro-dissection and phacoemulsification [22,24].

Titiyal et al. compared the morphology of CCIs in conventional phacoemulsification and FLACS using i-OCT; they noticed that a ragged slit morphology was a significant predictive factor for incision site DMD and it occurred more frequently during conventional surgery. Interestingly, the authors stated that all DMDs detected by i-OCT were also detectable under the operating microscope before stromal hydration; however, an increase in the extent of DMD or the occurrence of DMD after stromal hydration (which represented the phase in which the higher rate of DMD occurred—83.7% cases) were only detected by i-OCT. At any rate, all DMDs solved spontaneously in one month without requiring additional surgery [16]. The ability to detect an early subclinical DMD, an epithelial disruption, or a microtear in the inner or outer lip of the wound intraoperatively could be of great value for the surgeon not only to modify the subsequent steps of surgery but also to manage the early post-operative period [10].

The location of the Continuous curvilinear capsulorhexis (CCC) is critical for visual outcomes [30,31]. In conventional cataract surgery, the procedure is guided by the position of the PC and the LC, which are easily detected using a microscope; in FLACS, they can automatically be detected, together with an additional parameter called lens center. This represents very interesting data since the IOL center position will be similar to the center of the crystalline lens. A precisely sized and centered capsulotomy, enabled by this method, might improve predictability and control of the IOL placement reducing IOL tilting and decentration. Song et al. analyzed the relative location of and distance between the PC, the LC, and the lens center in patients who underwent FLACS. It was found that the PC was closer to the lens center than the LC whose X and Y coordinate position was significantly inferior and temporal compared to the PC [17,32].

Palanker et al. compared the size and the shape of laser capsulotomy to manual ones using a system combining FD-OCT with a femtosecond pattern scanning laser. They demonstrated that the former was characterized by size more similar to the intended one than the latter; moreover, they were more circular than manual ones [15].

Mastropasqua et al. analyzed the characteristics of capsulotomies obtained during two types of i-OCT guided FLACS platforms (Lensx and Lensar) and during a standard manual technique. Laser-made capsulotomies demonstrated significantly better circularity than the manual CCCs at seven days, their sizes were much more similar to the intended ones, and they showed greater IOLs centration than the manual group at all time points [18].

As for biometry images, optical opacity could also affect the quality of i-OCT images, leading to misleading analysis. During FLACS, precise detection of radiation sites is critical to correct the direction of spots and to avoid complications. Kurosawa et al. demonstrated that LT inspection could guide the surgeon in adjusting laser settings and avoiding posterior capsule breaks [14].

Moreover, real-time visualization of the trenching depth during phacoemulsification could be very useful for surgeons in training to decide the exact location to crack the nucleus during divide and conquer techniques [10].

Many authors have underlined the importance of i-OCT in complicated cases [33]. When dealing with white cataracts, the direct visualization of lens anatomical features through i-OCT could help anticipate the intraoperative dynamics of spontaneous milky fluid release, thus letting the surgeon be ready to deal with possible complications, especially during capsulorhexis [19].

For traumatic cataracts or PPCs, i-OCT could identify a capsular defect preventing further complications for the surgeon [20,29,34]. PPCs still represent a surgical challenge [35], due to the high incidence of posterior capsular break. To prevent it, hydrodissection is commonly avoided, consequently requiring greater manipulations during cortical clean-up and longer surgical time. In their study, Titiyal et al. evaluated morphological characteristics and intraoperative dynamics of PPCs with i-OCT, demonstrating that in the case of an intact posterior capsule homogenously spaced from the posterior polar opacity (called “type I PPC”) gentle hydrodissection could be safely performed. At any rate, the authors declared the preoperative AS-OCT features correlated with the intraoperative ones. Moreover, according to the authors, i-OCT use didn’t reduce the incidence of posterior capsule dehiscence compared to not i-OCT-guided surgeries [20].

I-OCT could also be helpful in patients with ectopia lentis, preventing further corneal endothelium damage during lens removal [36,37].

Juergens et al. reported i-OCT to be crucial for the implantation of a two-part brown iris diaphragm, because of the poor contrast between the anterior lens capsule margin and the brown implant [21].

Interestingly, i-OCT could detect the presence of direct intraoperative communication between Berger space and anterior chamber, which might lead to excessive fluid flow through this segment causing anterior displacement of the posterior capsule thus increasing the risk for a posterior capsular break and iris prolapse. Anisimova et al. showed that i-OCT could identify the presence of lens micro fragments and cellular material within the Berger space for the discontinuity of the zonules and Wieger ligament. with a higher sensitivity than postoperative OCT. Furthermore, they hypothesized that Wieger ligament detachment was associated with increased zonular permeability. This observation could be useful to clarify the mechanism of acute aqueous misdirection syndrome also known as acute rock-hard eye syndrome (AIRES) [6].

Although i-OCT might represent a helpful and not invasive tool, its application in clinical practice presents several limitations for cataract surgery. Firstly, intraoperative measurements are still time-consuming. Secondly, OCT-friendly instruments to reduce shadowing and integrated calipers are still lacking [10,13]. Moreover, total cataracts or extremely dense nuclear sclerosis reduce the ability of i-OCT to visualize the posterior capsule [19,20]. Finally, the analysis of the intraoperative images is not automatic, and it is still influenced too much by the insights of the observer.

## 5. Conclusions

In summary, the use of i-OCT in cataract surgery may represent a useful tool for novel surgeons approaching phacoemulsification, but also for expert ones for teaching purposes and to plan and manage complicated cases. I-OCT could also be employed to avoid refractive errors or intraoperative complications in patients with white cataracts or PPCs and to identify micro fragments in the Berger space. However, only a few studies have shown a sufficient grade or level of evidence and some limitations have been pointed out. Prospective studies would be ideal to pursue the question of the advantages of the use of i-OCT in cataract surgery and the factors or conditions that may indicate its use. 

## Figures and Tables

**Table 1:** Characteristics, quality, and level of evidence of the included studies and features of the anterior segment intraoperative optical coherence tomography (i-OCT) cited by these articles.

Author	Year	Study Design	Study Sample	Type of Surgery (n° of Eyes)	Type of Cataract (n°)	Intraoperative OCT (i-OCT) Specifications	Ocular Evaluation	Results	Grade ^1^	Level ^2^
Das S.	2016	Prospective study (P)	38 eyes (E)	Microincision cataract surgery (MICS) (28); femtosecond laser assisted cataract surgery (FLACS) (10)	Posterior polar cataracts (PPCs) (3); mature intumescent cataracts (2); nuclear cataracts (N) grade 2–3 (33).	RESCAN^TM^ 700 (Carl Zeiss Meditec)	To describe the role of i-OCT in MICS and FLACS, focusing on wound assessment, capsulorhexis, hydroprocedures, nucleus management, intraocular lens (IOL) assessment	I-OCT could be useful for assessing wound morphology, deciding the adequate depth of trenching, and detecting intraocular lens (IOL) position.	Low	4
Tañá-Sanz P.	2021	P	102 E	FLACS	Not specified (NS)	Catalys (Johnson & Johnson Vision)	To compare different parameters obtained by i-OCT (before starting surgery) and preoperative OCT biometry.	Measurements provided by Catalys, IOLMaster 700 (Carl Zeiss Meditec) and Anterion (Heidelberg Engineering) are significantly different.	Low	4
Waring G.O. 4th	2020	Retrospective study (R)	293 E	FLACS (235); femtosecond laser-assisted refractive lens exchange (58)	NS	Catalys	To analyze the existing relationship among i-OCT-derived lens parameters, biometry, and age.	Commonly available biometric data couldn’t predict i-OCT-derived lens parameters such as lens diameter and lens volume.	Moderate	4
Hirnschall N.	2013	P	70 E	MICS	NS	Visante (Carl Zeiss Meditec)	To analyze the potential role of i-OCT-derived parameters (acquired after crystalline lens removal) in prediction of postoperative IOL position	I-OCT measurement of anterior capsule position after capsular tension ring (CTR) insertion was a better predictor of the early postoperative IOL position compared with preoperative data.	Low	4
Kurosawa M.	2021	R	1070 E	FLACS	NS	Catalys	To study whether comparing preoperatively and intraoperatively acquired lens thickness (LT) could help in preventing surgical complications during nuclear laser irradiation in FLACS.	LT inspection could be useful to reduce inappropriate posterior capsule detection cases and consequently misdirected femtosecond laser spots.	Low	4
Palanker D.V.	2010	P	30 patients	MICS (30); FLACS (29)	N grade 1 to 4	Frequency-domain OCT (FD-OCT) model integrated on microscope	To develop a model of i-OCT-guided FLACS and to compare it with MICS, focusing on the capsulotomy step.	Capsulotomies performed by OCT-guided femtosecond lasers were characterized by sizes and shapes which were more similar to the intended ones than manual capsulorhexis.	Low	4
Titiyal J.S.	2018	P	129 E	MICS (77); FLACS (52)	NS	RESCAN^TM^ 700	To evaluate the morphology of clear corneal incisions (CCIs) and their impact on Descemet membrane detachment (DMD).	A ragged morphology of CCIs was associated with a higher incidence of DMD; only i-OCT could detect an increase in its size or its development after stromal hydration.	Low	4
Song V.K.	2019	R	35 (E)	FLACS	NS	Catalys	To study the anatomical overlap between the pupil center (PC), the limbal center (LC) and the lens center, in order to guide capsulotomy.	The PC was nearer to the lens center than the LC.	Very low	4
Mastropasqua L.	2014	P	90 E	Lensx (Alcon Laboratories) FLACS (30); Lensar (Lensar) FLACS (30); MICS (30).	NS	Lensx; Lensar	To compare capsulotomies obtained with FLACS with manual capsulorhexis.	FLACS capsulotomies were greater than manual ones, determining a more precise IOL centration.	Moderate	3
Titiyal J.S.	2020	P	50 E	MICS	White cataracts	RESCAN^TM^ 700	To analyze white cataract morphology and intraoperative dynamics, focusing on capsulorhexis.	I-OCT permitted the identification of four types of white cataracts, based on their anatomical characteristics and surgical behavior during capsulorhexis, helping the surgeon dealing with rhexis’ extension-related complications.	Low	4
Titiyal J.S.	2020	P; R	112 E	MICS	PPCs	RESCAN^TM^ 700	To evaluate the morphology of PPCs, intraoperative dynamics of the posterior capsule and the occurrence of posterior capsular dehiscence.	I-OCT could help in detecting those PPCs which could undergo safe hydrodissection.	Low	4
Anisimova N.S.	2020	P	28 videos	MICS (13); FLACS (15)	N	RESCAN^TM^ 700	To identify the presence of incomplete vitreolenticular adhesion, immediately after IOL implantation.	I-OCT permitted the identification of undesired particles into Berger’s space with a higher sensitivity than post-operative OCT.	Very low	4
Juergens L.	2021	P	4 E	Standard phacoemulsification combined with iris diaphragm implantation	NS	EnFocus Ultra-Deep OCT (Leica Microsystems)	To assess when the use of i-OCT could be relevant for intra- operative procedures.	I-OCT was crucial for the implantation of a two-part brown iris diaphragm, because of the poor contrast between the anterior lens capsule margin and the brown implant.	Very low	5

^1^ Quality of evidence according to the Grading of Recommendations Assessment, Development and Evaluation (GRADE) system [9]. ^2^ Level of evidence according to the Oxford Centre for Evidence-Based Medicine (OCEM) 2011 guidelines [8].

## Data Availability

Data are available on reasonable request by the corresponding author.
